# Biological formation of ethylene

**DOI:** 10.1039/d3cb00066d

**Published:** 2023-07-10

**Authors:** Robert P. Hausinger, Simahudeen Bathir J. S. Rifayee, Midhun G. Thomas, Shramana Chatterjee, Jian Hu, Christo Z. Christov

**Affiliations:** a Department of Microbiology and Molecular Genetics, Michigan State University East Lansing Michigan 48824 USA; b Department of Biochemistry and Molecular Biology, Michigan State University East Lansing Michigan 48824 USA hausinge@msu.edu; c Department of Chemistry, Michigan Technological University Houghton Michigan 49931 USA; d Department of Chemistry, Michigan State University East Lansing Michigan 48824 USA

## Abstract

This review summarizes the structures, biochemical properties, and mechanisms of two major biological sources of ethylene, the ethylene-forming enzyme (EFE) and 1-aminocyclopropane-1-carboxylic acid (ACC) oxidase (ACCO). EFE is found in selected bacteria and fungi where it catalyzes two reactions: (1) the oxygen-dependent conversion of 2-oxoglutarate (2OG) to ethylene plus three molecules of CO_2_/bicarbonate and (2) the oxidative decarboxylation of 2OG while transforming l-arginine to guanidine and l-Δ^1^-pyrroline-5-carboxylic acid. ACCO is present in plants where it makes the plant hormone by transforming ACC, O_2_, and an external reductant to ethylene, HCN, CO_2_, and water. Despite catalyzing distinct chemical reactions, EFE and ACCO are related in sequence and structure, and both enzymes require Fe(ii) for their activity. Advances in our understanding of EFE, derived from both experimental and computational approaches, have clarified how this enzyme catalyzes its dual reactions. Drawing on the published mechanistic studies of ACCO and noting the parallels between this enzyme and EFE, we propose a novel reaction mechanism for ACCO.

## Abstract 2

Ethylene formation by the ethylene-forming enzyme (EFE) and 1-aminocyclopropane-1-carboxylate oxidase (ACCO).

## Graphical abstract

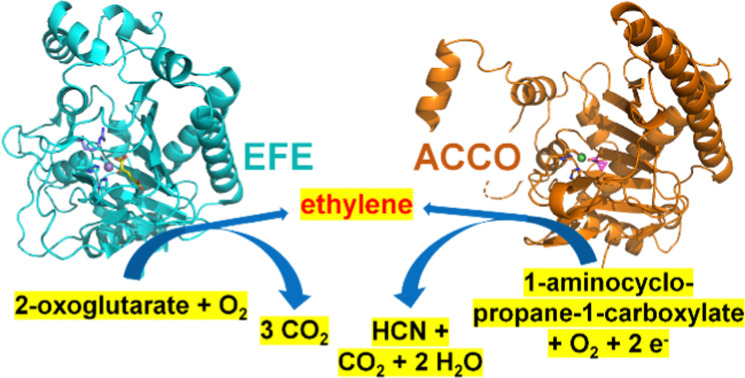

## Introduction

Ethylene is produced at a vast scale (>150 million metric tons in 2016) from natural gas or petroleum and is widely used in the chemical industry as a building block for the synthesis of plastics and other materials. The industrial production of this gas leads to the emission of high levels of carbon dioxide and other greenhouse gases,^1^ which has spurred interest in exploring alternative methods to make ethylene in a more sustainable manner from renewable sources.^2–4^ Here, we describe four different approaches used in biology for the enzymatic synthesis of ethylene, focusing on the first two reactions.

**Fig. 1:**
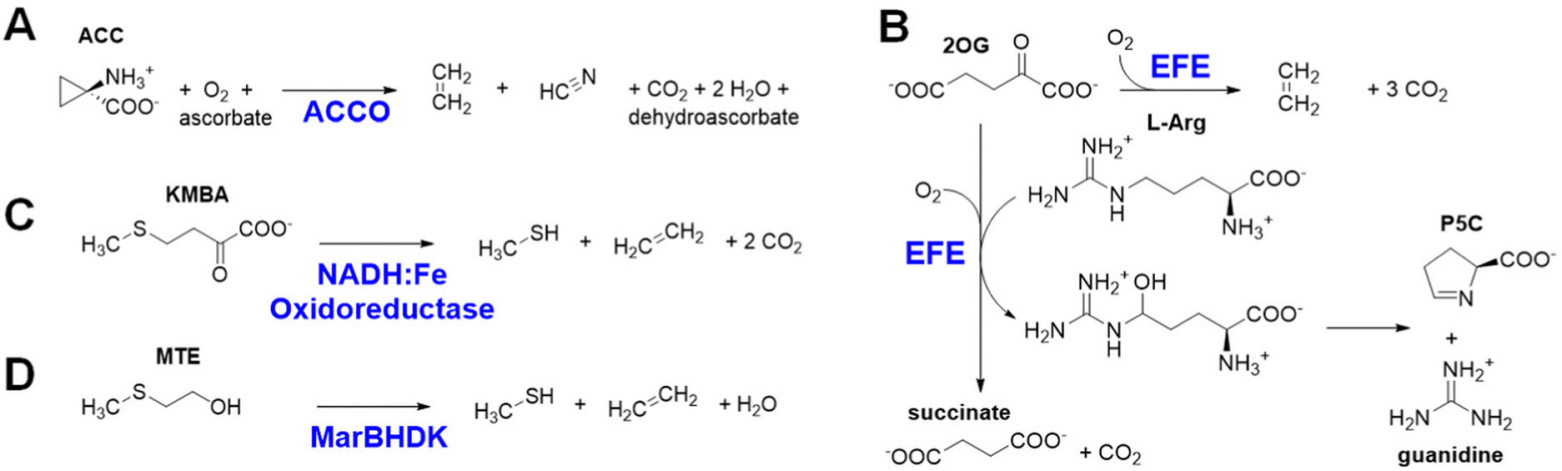
Four biological processes for ethylene production. (A) ACCO conversion of ACC to ethylene, hydrogen cyanide, and carbon dioxide. (B) EFE oxidative transformation of 2OG to ethylene and three molecules of carbon dioxide in the presence of l-Arg, and its C5 hydroxylation of l-Arg as 2OG undergoes oxidative decarboxylation to form succinate. (C) NADH:Fe(iii) oxidoreductase promoted decomposition of KMBA to form ethylene, methanethiol, and two molecules of carbon dioxide. (D) MarBHDK reduction of MTE to ethylene, methanethiol, and water.

The most well-known biological method to make ethylene occurs in some fungi and many plants that use it as a hormone to regulate growth and development.^5^ The plant pathway for ethylene synthesis begins with the conversion of l-methionine to *S*-adenosyl-l-methionine (SAM) by *S*-adenosyl-l-methionine synthetase.^6^ SAM is transformed into 1-aminocyclopropane-1-carboxylic acid (ACC) by the pyridoxal-5′-phosphate-dependent enzyme ACC synthase.^7^ Finally, ACC is converted to ethylene, carbon dioxide, and hydrogen cyanide by ACC oxidase (ACCO) (Fig. 1A).^8^ Plants sense the levels of ethylene in their environment using a copper-containing sensor protein,^9,10^ resulting in fruit ripening and other effects.

Selected bacteria and fungi have a distinct enzyme called the ethylene-forming enzyme (EFE) that exhibits two activities (Fig. 1B).^11,12^ Its primary reaction is to convert 2-oxoglutarate (2OG) to ethylene and three molecules of carbon dioxide/bicarbonate (CO_2_) in an oxidative reaction that requires, but does not transform, the amino acid l-Arg. The secondary reaction catalyzed by EFE is to hydroxylate C5 of l-Arg, creating an unstable species that decomposes to guanidine and l-Δ^1^-pyrroline-5-carboxylic acid (P5C), while converting 2OG to succinate and carbon dioxide. EFE increases the virulence of plant pathogens,^13^ perhaps because the phytohormone causes metabolic imbalances in the plant, thus weakening cellular defenses.

A third biological source of ethylene, albeit at low levels, has been reported in cell extracts of *Escherichia coli* and *Cryptococcus albidus* and shown to involve the product of l-methionine transamination, 2-keto-4-methylthiobutyric acid (KMBA), and NADH:Fe(iii)-EDTA (NADH:Fe) oxidoreductase (Fig. 1C).^14,15^ The enzyme from *C. albidus* was purified and shown to reduce two Fe(iii) to Fe(ii) as NADH was oxidized to NAD^+^. The activity of NADH:Fe oxidoreductase towards KMBA was suggested to involve hydroxyl radical formation with the products tentatively identified as ethylene, methanethiol, and two carbon dioxide,^15,16^ but the detailed chemical mechanism was not examined.

The most recently identified biological source of ethylene is associated with a wide variety of microorganisms that inhabit anaerobic environments.^17,18^ These microbes metabolize 2-(methylthio)ethanol (MTE), derived in a series of reactions from 5′-methylthioadenosine, by a reductive process using a nitrogenase-like reductase (MarBHDK) to form ethylene, methanethiol, and water (Fig. 1D). The enzyme has not been purified and its mechanism of catalysis is unknown. This system and that catalyzed by the NADH:Fe oxidoreductase will not be further discussed.

## Sequence and structure relationships of EFE and ACCO

**Fig. 2:**
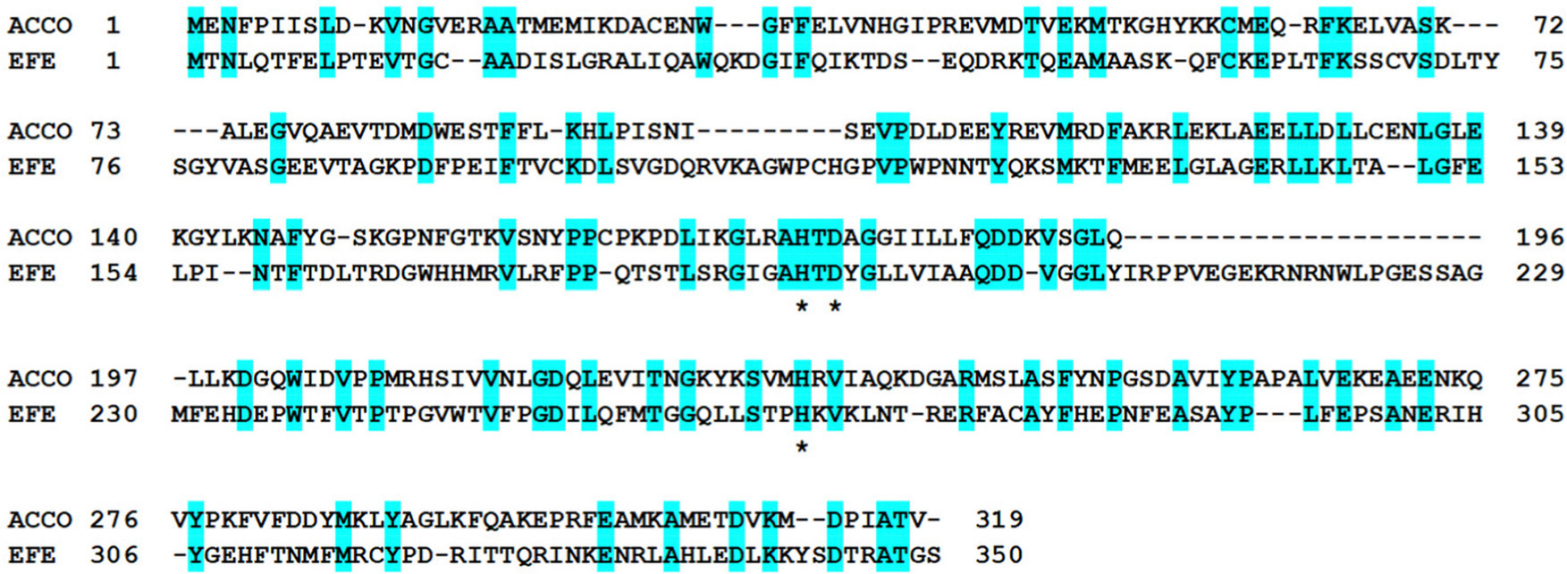
Sequence comparison of *Petunia x hybrida* ACCO (top) and *Pseudomonas savastanoi* pv. *phaseolicola* strain PK2 EFE (bottom). Identical residues are highlighted in cyan. The metal-binding ligands are indicated by asterisks.

**Fig. 3:**
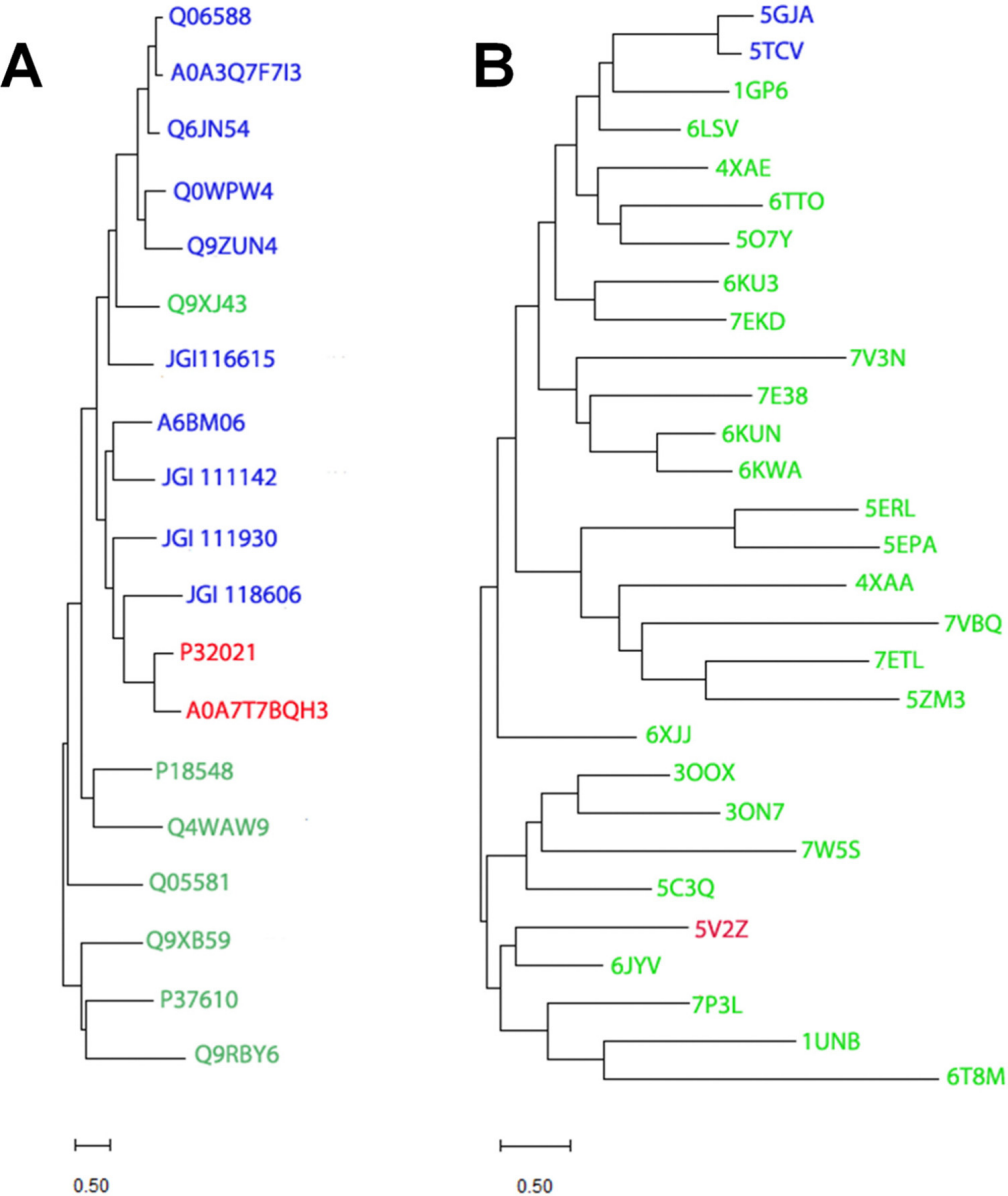
Phylogenetic and structural dendrograms comparing selected sequences and structures of EFE, ACCO, and related Fe(ii)/2OG-dependent oxygenases. (A) A maximum likelihood phylogenetic tree constructed using the Poisson correction model and the MUSCLE alignment plugin in MEGA11.^24^ Gaps and missing data were eliminated using the complete deletion option. UniprotKB or JGI numbers identify sequences for EFE (red) from strain PK2 and *Penicillium digitatum* (P32021 and A0A7T7BQH3, respectively), ACCO (blue) including type 1 from *Arabidopsis thaliana*, *Solanum lycoperscim*, and *Zea mays* (Q06588, A0A3Q7F7I3, and Q6JN54, respectively), types 3 and 2 from *A. thaliana* (Q0WPW4 and Q9ZUN4 respectively), and type 4 from *Dictyostelium mucoroides* (A6BM06) and *Volvierella volvacea* (JGI 116615, JGI 111142, JGI 111930, and JGI118606), and other Fe(ii)/2OG oxygenases (green), including hyoscyamine 6-β-hydroxylase from *Atropa belladonna* (Q9XJ43), deacetoxycephalosporin C synthase from *Streptomyces clavuligerus* (P18548), verruculogen synthase from *Aspergillus fumigatus* (Q4WAW9), clavaminate synthase 1 from *Streptomyces clavuligerus* (Q05581), (5R)-carbapenem-3-carboxylate synthase from *Pectobacterium carotovorum* (Q9XB59), taurine/2-oxoglutarate dioxygenase from *Escherichia coli* (P37610), and L-threonyl-(threonyl carrier protein) 4-chlorinase from *Pseudomonas syringae* pv. *syringae* (Q9RBY6). (B) Hierarchical clustering of structures denoted by PDB access codes. Structures related to strain PK2 EFE (red, 5V2Z) were identified using DALI^25^ with a Z-score up to 12 and clustered using MEGA11.^24^ The two reported ACCO structures (5GJA and 5TCV) are shown in blue. Other Fe(ii)/2OG oxygenases (green) include anthocyanidin synthase from *A. thaliana* (1GP6),), feruloyl-CoA 6-hydroxylase from *A. thaliana* (4XAE), hyoscyamine 6-hydroxylase from *Datura metel* (6TTO), thebaine-6-*O*-demethylase from *Papaver somniferum* (5O7Y), gibberellin 2β-hydroxylase and gibberellin 3β-hydroxylase from *Oryza sativa* (6KU3 and 7EKD, respectively), deoxypodophyllotoxin synthase from *Sinopodophyllum hexandrum* (7E38), dioxygenase for auxin oxidation from *O. sativa* and *A. thaliana* (6KUN and 6KWA, respectively), SnoN epimerase from *Streptomyces nogalater* (5ERL), SnoK carbocyclase from *S. nogalater* (5EPA), the TlxIJ enzyme in meroterpenoid biosynthesis in *Talaromyces purpureogenus* (7VBQ), FtmOx1 from *Aspergillus fumigatus* (7ETL), TropC involved in tropolone biosynthesis in *Talaromyces stipitatus* (6XJJ), a halogenase from *Actinomadura* sp. ATCC 39365 (7W5S), thymine 7-hydroxylase from *Neurospora crassa* (5C3Q), isopenicllin N synthase from *Emericella nidulans* (7P3L), deacetoxycephalosporin C synthase from *S. clavuligerus* (1UNB), prolyl hydroxylase from *Dictyostelium discoideum* (6T8M), and other enzymes from various sources with still undefined functions (6LSV, 7V3N, 4XAA, 5ZM3, 3OOX, 3ON7, and 6JYV). The bootstrap method was used for each panel with 1000 replicates.

Despite catalyzing quite divergent chemical reactions (Fig. 1A and B), the sequences of EFE and ACCO are related (Fig. 2), as illustrated using the best studied representatives of these enzymes, EFE from *Pseudomonas savastanoi* (also known as *P. syringae*) pv. *phaseolicola* strain PK2 (hereafter denoted strain PK2 EFE)^19^ and ACCO from *Petunia x hybrida*.^20^ The protein sequences are over 27% identical, including the positions of the metal binding residues. Notably, both enzymes are dependent on ferrous ions for activity. The sequence relationship between EFE and ACCO proteins has been noted by others previously,^21^ and is reflected in a targeted phylogenetic tree that includes two representatives of EFE, the reported four types of ACCO proteins,^22,23^ and selected other Fe(ii)/2OG-dependent oxygenases (Fig. 3A). EFE and type 4 ACCO sequences (derived from mushrooms and slime molds) are more closely related whereas similarities between EFE and types 1–3 ACCO sequences (from plants) are more distant, but still closer than several other well studied Fe(ii)/2OG oxygenases.

**Fig. 4:**
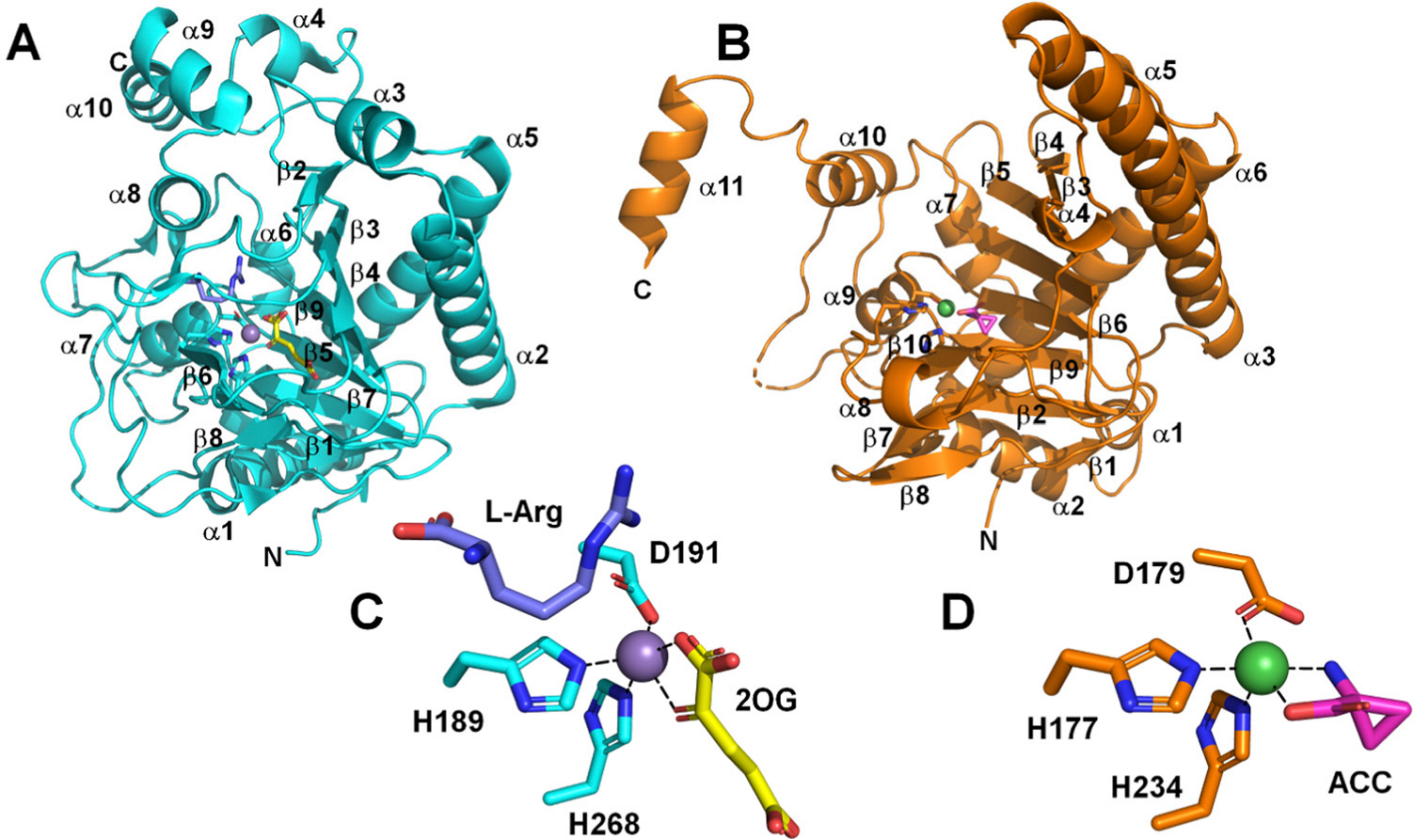
Comparison of EFE and ACCO structures. Cartoon depiction of (A) strain PK2 EFE (cyan, PDB: 5V2Y) and (B) ACCO (sienna, PDB: 5TCV). (C) Active site of EFE with 2OG (yellow), l-Arg (dark blue), and the metal-binding ligands shown as sticks, and with inactive Mn (substituting for Fe) as a purple sphere. (D) Active site of ACCO with ACC (magenta) and the metal binding ligands shown as sticks, and with inactive Ni (also substituting for Fe) as a green sphere.

Not surprisingly, the structures of strain PK2 EFE^26–28^ and ACCO^29^ also are similar with both enzymes exhibiting a double-stranded β-helix (DSBH or jellyroll) fold that is common to all members of the 2OG oxygenases for which crystal structures are available^30,31^ and they have comparable positions for the metals and their ligands (Fig. 4). PK2 EFE (PDB: 5V2Y) forms a DSBH core with nine β-strands, six from the major β-sheet and three from the minor β-sheet (Fig. 4A). This core is surrounded and stabilized by 10 α-helices. Residues 80–93 for PK2 EFE act as a “lid” that covers or shields the active site from the solvent. This lid may help maintain the hydrophobic environment surrounding 2OG or function in substrate binding; its precise role is not defined. The ACCO (PDB: 5TCV) main chain contains eleven α helices and twelve β strands, eight of which (β-3 to β-10) form the distorted DSBH (Fig. 4B). It too has a loop (residues 160–220) that includes a lid (residues 199–202) over the active site with no established function. For ACCO, most of the α helices are located in the N-terminal (α-1 to α-6) and C-terminal (α-8 to α-11) sides of the DSBH. A supporting matrix is formed through N-terminal helices on one face of the DSBH. The open-faced active site is located at one end of the jellyroll. The EFE and ACCO structures exhibit the same overall fold with an RMSD of ∼2 Å. Indeed, these enzymes are structurally more related to each other than to many other members of the Fe(ii)/2OG oxygenases (Fig. 3B). The active site structure of EFE (Fig. 4C) resembles that of many other Fe(ii)/2OG-dependent oxygenases, with 2OG chelating the metal (when l-Arg is present) and positioned with its 2-keto oxygen opposite the carboxyl residue and the C1 carboxylate opposite one of two histidyl residues. The binding of ACC to ACCO (Fig. 4D) similarly has a chelate structure, in this case with the carboxylate group opposite the carboxyl residue and the amine opposite a histidyl residue. Crystallographic structures of Fe(ii) oxygenases and oxidases often substitute other metal ions [*e.g.*, Mn(ii), Ni(ii), and Co(ii)] for Fe(ii) to avoid the oxygen reactivity of the native metal ion; however, the resulting structures are thought to be faithful mimics of the Fe(ii)-bound enzymes.

## Properties of EFE in the absence of oxygen

**Fig. 5:**
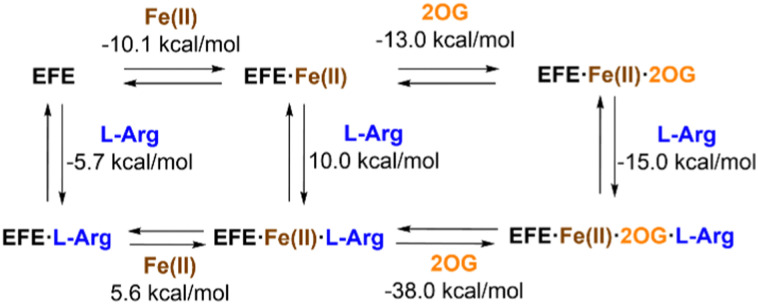
Enthalpy changes associated with the binding of Fe(ii), 2OG, and l-Arg to strain PK2 EFE.

The interactions of EFE from strain PK2 with Fe(ii), 2OG, and l-Arg were examined by isothermal titration calorimetry and differential scanning calorimetry.^32^ The addition of each of these components enhances the stability of EFE compared to the apoprotein, with 2OG and l-Arg exhibiting cooperativity in binding. The differences in enthalpy changes (*i.e.*, *δ*Δ*H*) associated with the binding of these components is shown in Fig. 5. Of particular interest, the enthalpy value measured for 2OG binding to EFE·Fe(ii) (−13.0 kcal mol^−1^) differs significantly from that for another well-characterized Fe(ii)/2OG oxygenase (TauD) that exhibits an enthalpy-driven process (−30.1 kcal mol^−1^),^33^ indicating a distinction associated with 2OG binding to EFE.

**Fig. 6:**
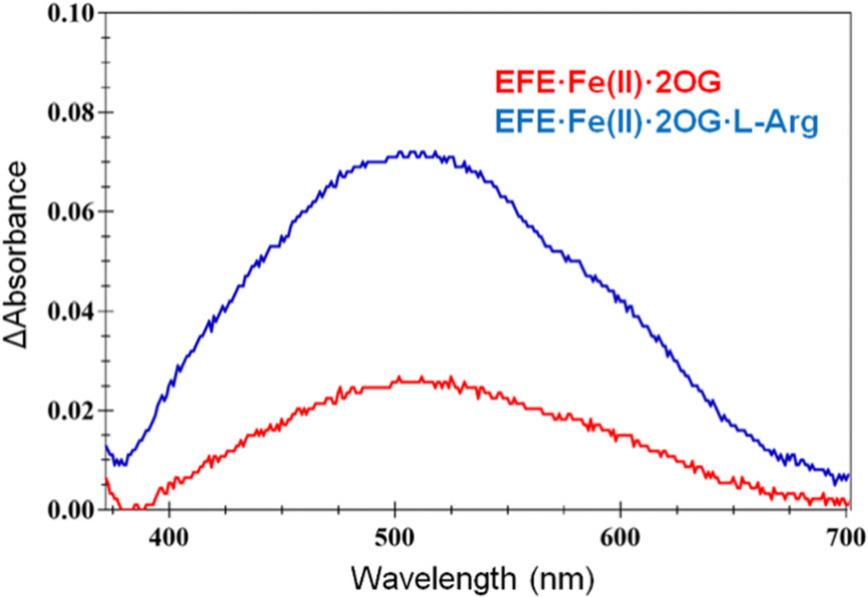
UV-visible difference spectra for EFE·Fe(ii)·2OG (red) and EFE·Fe(ii)·2OG·l-Arg, with the spectrum of EFE·Fe(ii) subtracted. The concentrations were 226 μM protein, 1 mM Fe(ii), 2.4 mM 2OG, and (when present) 2.7 mM l-Arg.

The interactions of 2OG and l-Arg with strain PK2 EFE·Fe(ii) were probed by anaerobic UV-visible spectroscopy.^34^ The EFE apoprotein and the Fe(ii)-bound states of the enzyme possess no electronic transitions other than those contributing to its 280 nm absorption. Upon addition of 2OG, however, a very weak absorption at ∼515 nm is observed (*ε* = 114 M^−1^ cm^−1^), and this intensity increases ∼3-fold (*ε* = 314 M^−1^ cm^−1^) and develops a clear shoulder in the presence of l-Arg (Fig. 6). These features have been assigned in another system as arising from metal-to-ligand charge-transfer transitions associated with chelation of Fe(ii) by 2OG,^35^ but the dramatic effect of l-Arg on the intensity of this absorption is unprecedented among members of this enzyme family. These spectroscopic results are consistent with EFE·Fe(ii)·2OG being a mixture of states that includes both monodentate- and bidentate-bound 2OG. This situation accounts for the small enthalpy change measured for 2OG binding to EFE·Fe(ii) cited earlier. The binding of l-Arg shifts the equilibrium to the fully chelated species.

**Fig. 7:**
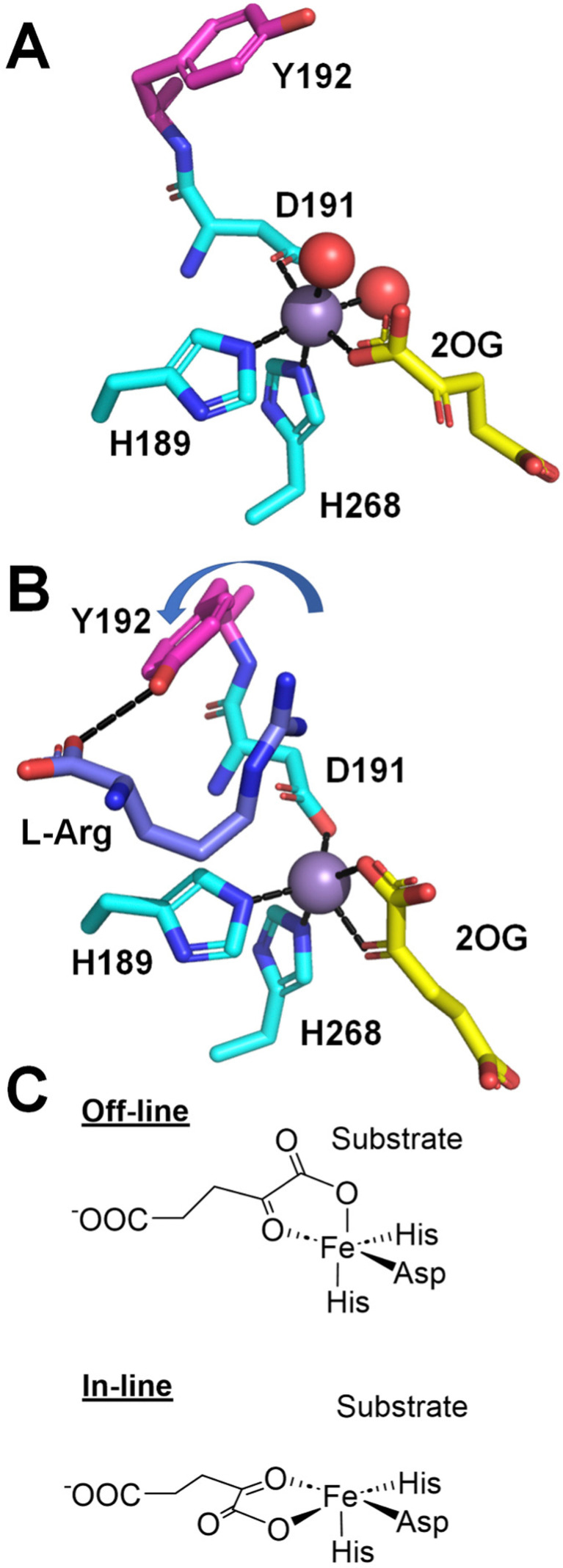
Structural changes at the active site induced by the binding of l-Arg. (A) EFE·Mn(ii)·2OG (PDB: 5V2X). (B) EFE·Mn(ii)·2OG·l-Arg (PDB: 5V2Y). l-Arg binding leads to a shift from monodentate to bidentate binding of 2OG, a switch in the oxygen atom of D191 that coordinates the metal, and a flip of Y192 (depicted with red carbon atoms) that creates a twist in the D191-Y192 peptide bond. (C) Comparison of off-line (as in B) and in-line binding modes of 2OG.

Direct evidence that strain PK2 EFE displays altered 2OG binding modes for the EFE·Mn(ii)·2OG and EFE·Mn(ii)·2OG·l-Arg complexes was obtained by crystallographic analysis of the structures.^27^ (A full list of the available crystal structures for EFE is provided in Table 1). In the EFE·Mn(ii)·2OG complex (Fig. 7A), 2OG coordinates the Mn(ii) in a partial monodentate manner, allowing for two water molecules to remain bound to the metal ion. Upon binding l-Arg (Fig. 7B), the 2OG adopts a chelate structure, and both water molecules dissociate, thus providing a position for oxygen to bind at the sixth coordination site of the metal. Of additional interest, the D191 ligand alters its position to coordinate the metal with the alternate oxygen atom of the carboxylate. Furthermore, Y192 flips its orientation and forms a hydrogen bond with the carboxyl group of l-Arg, leading to a twisted peptide bond between D191 and Y192. Another key point to note about Fig. 7B is that the metal coordination site for binding dioxygen is *trans* to H189 and not pointed toward l-Arg. This geometry has been termed off-line, whereas in many members of this enzyme family, structural studies have revealed an in-line geometry with the oxygen binding site pointed toward the substrate (Fig. 7C).^36^

**Table 1:** Structures reported for EFE

Enzyme^a^	Metal	Ligand(s)	Resolution (Å)	PDB Code	Ref.
WT	Mn	Bis-Tris-propane	1.55	5SQL	26
WT	Fe	NOG·l-Arg	1.08	5LUN	26
WT	None	None	2.06	5V2U	27
WT	Ni		3.04	5V2V	27
WT	Mn	2OG	1.85	5V2X	27
WT	Mn	2OG·l-Arg	1.43	5V2Y	27
WT	Mn	2-Oxoadipic acid·l-Arg	1.23	5V2Z	27
WT	Mn	2OG·N-hydroxy-l-Arg	1.17	5VKA	27
WT	Mn	2OG·argininamide	1.14	5VKB	27
WT	Mn	l-Arg	2.45	5V31	27
WT	Mn	Malate	1.49	5V32	27
WT	Mn	Tartaric acid	1.23	5V2T	27
WT	Mn	Malate·l-Arg	1.48	5V34	27
Y306A	Mn	2OG	1.12	6CF3	Unpublished
WT	Mn	2OG·canavanine	1.13	6CBA	Unpublished
WT	Fe	2OG·l-Arg	1.83	6VP4	28
D191E	Fe	2OG·l-Arg	1.97	6VP5	28

a In all cases, the enzyme was derived from *Escherichia coli* cells expressing the gene encoding *Pseudomonas savastanoi* (alternatively called *P. syringae*) *pv. phaseolicola* strain PK2, either the wild-type (WT) version or a variant.

## EFE reactivity with oxygen

**Fig. 8:**
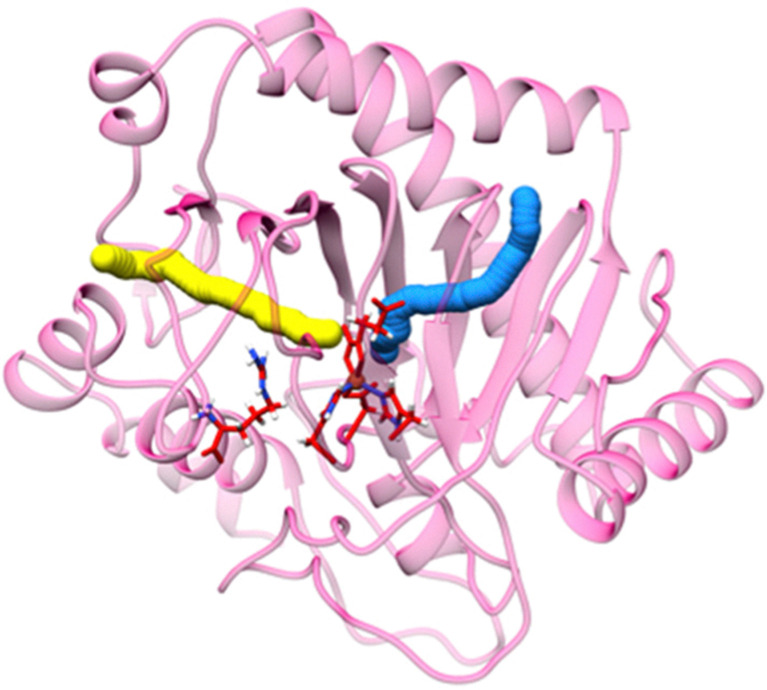
Computed tunnels for oxygen access to the EFE active site. Tunnel-1 is represented in blue, and tunnel-2 is shown in yellow. The red sticks depict l-Arg, 2OG, and the three metal-binding side chains.

Molecular dynamics (MD) simulations of dioxygen diffusion dynamics using the EFE crystal structure predicted the existence of two tunnels allowing oxygen to access Fe(ii) at the active site (Fig. 8).^37^ The primary tunnel-1 (18.9 Å in length with a bottleneck radius of 1.15 Å) allows oxygen to approach the metallocenter *trans* to H189 and would directly yield an off-line Fe(iii)-superoxo state directed away from l-Arg. In contrast, the secondary tunnel-2 (22.4 Å in length and with a bottleneck radius of 1.1 Å) would allow oxygen to approach *trans* to H268 but would require 2OG to undergo a conformational shift of the C1 carboxylate (from opposite H268 to opposite H189) to form an in-line Fe(iii)-superoxo state that points toward l-Arg. While it is plausible that the two oxygen access tunnels are correlated with the ethylene-forming *versus*l-Arg hydroxylation reactivities of EFE (Fig. 1B), quantum mechanics/molecular mechanics (QM/MM) calculations indicate the transformation of an off-line EFE·Fe(ii)·2OG·l-Arg complex to an in-line EFE·Fe(ii)·2OG·l-Arg complex is energetically unfavorable with an energy barrier of 21.8 kcal mol^−1^ and the latter complex is endothermic by 20.9 kcal mol^−1^.^38^ Furthermore, simulation of the free energy surface for dioxygen approaching Fe(ii) with an in-line configuration reveals the in-line Fe(iii)-superoxo complex is not energetically stable.^37^ The switching of 2OG coordination from bidentate to monodentate could be a factor that destabilizes the formation of the in-line Fe(iii)-superoxo complex. Thus, oxygen likely gains access to the EFE active site using only tunnel-1 and forms the off-line Fe(iii)-superoxo state for all subsequent reactivities. Substrate does not form a part of the dioxygen diffusion tunnel and is not involved in stabilizing the Fe(iii)-superoxo intermediate.

The two major reactions of EFE, ethylene formation and l-Arg hydroxylation, initiate from the same activated oxygen state but are not linked.^34^ For example, 2-oxoadipate (2OA), an alternative 2-oxo-acid, undergoes oxidative decarboxylation (forming glutaric acid and carbon dioxide) while hydroxylating l-Arg, but it does not decompose and form ethylene. Additionally, the enzyme does not metabolize N^γ^-hydroxy-l-Arg, but in the presence of this compound EFE generates ethylene from 2OG. Site-directed mutagenesis studies also support a lack of coupling between the two reactions. For example, D191E, A198V, F283A/R/V/W, and E285A/Q variants all allow for some l-Arg hydroxylation activity, as shown by the production of P5C, while essentially eliminating the ethylene-forming activity.^27,28^ The original description of the two reactions for EFE stated that ethylene formation *versus*l-Arg hydroxylation occurred in a 2 : 1 ratio,^11^ but more recent studies have demonstrated the ratio varies somewhat with experimental conditions, and this ratio ranges from 2 : 1 to over 4 : 1.^28,34^

**Fig. 9:**
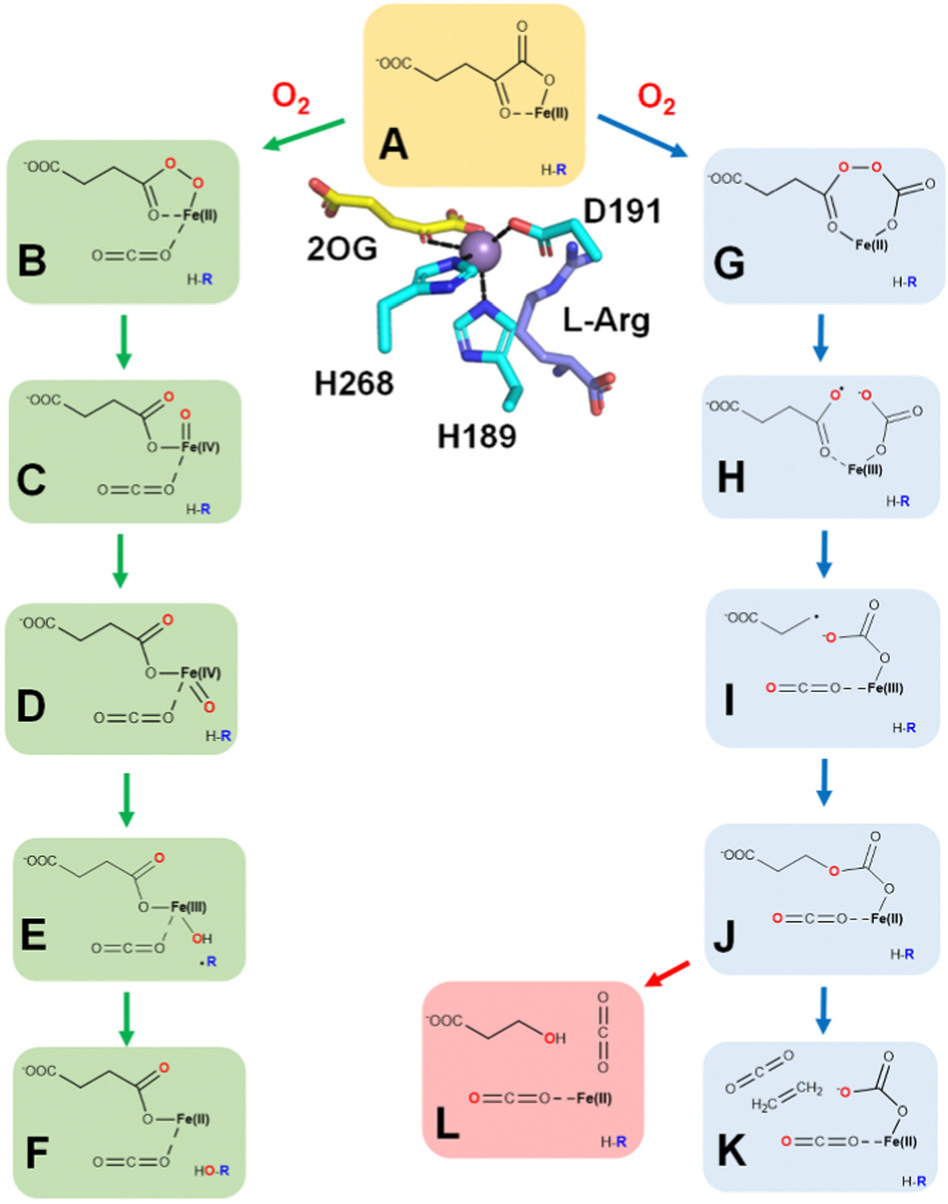
Proposed reaction mechanisms of EFE. (A) EFE·Fe(ii)·2OG with an open coordination site opposite of H189. (B–F) States in the pathway of l-Arg hydroxylation as 2OG undergoes oxidative decarboxylation. (G–K) States in the pathway of ethylene biosynthesis. (L) Very minor side reaction that converts 2OG to 3-hydroxypropionate. H-R is the substrate l-Arg.

The minor activity of EFE, hydroxylation at C5 of l-Arg, resembles the hydroxylation processes associated with a large number of Fe(ii)/2OG-dependent oxygenases,^39–43^ and is likely to occur by steps illustrated on the left of Fig. 9. Oxygen binds to the open coordination site (*trans* to H189) of EFE·Fe(ii)·2OG·l-Arg (state A, illustrated both in ChemDraw and crystal structure views) to form an initial off-line Fe(iii)-superoxo state (not shown). Attack of the superoxo group on C2 of 2OG results in its decarboxylation and formation of Fe(ii)-peroxysuccinate (state B). A comparable state has been crystallographically identified in VioC, an Fe(ii)/2OG oxygenase that hydroxylates C3 of l-Arg.^44^ The O–O bond of the succinylperoxy state is cleaved to form a succinate-bound Fe(iv)-oxo (ferryl) intermediate (state C). Evidence for the ferryl state was derived from stopped-flow UV-visible (SF-UV-vis) spectroscopy, with the changes in absorption at 318 nm used to monitor its rates of formation and decay, and by its characteristic Mössbauer parameters (*δ* = 0.26 mm s^−1^, Δ*E*_Q_ = 0.96 mm s^−1^).^28^ Furthermore, the use of 5,5-[^2^H]-l-Arg reduced by 16-fold the rate of decay for the ferryl state, as expected for the key hydrogen atom transfer (HAT) step.^28^ The ferryl oxygen atom initially points away from the l-Arg and is presumed to undergo a “ferryl flip” to become directed towards the substrate (state D). HAT creates a substrate radical and the Fe(iii)-hydroxide intermediate (state E). Hydroxyl radical rebound completes the hydroxylation reaction to regenerate the Fe(ii) state (state F) that exchanges its non-protein ligands with water while the hydroxylated l-Arg decomposes to guanidine and P5C.

Computational analyses are consistent with the sequence of states shown in the left half of Fig. 9 during the l-Arg hydroxylation reaction of EFE.^38,45,46^ MD simulation of the off-line Fe(iii)-superoxo complex demonstrated the existence of two conformations of l-Arg, as reported for a crystal structure of EFE (5LUN).^26^ In one conformation, C5 is closer to the Fe center, whereas, in the other conformation, the Fe–C5 distance is longer.^38^ The population of the two conformations in the MD simulation was 11.7% and 88.3%, respectively. The first conformation of l-Arg was predominantly stabilized by the electrostatic interaction of R171 with the guanidinium group. Weakening of this interaction in the R171A variant drives the transition to the second conformation. The second conformation is stabilized by an interaction of the amino group of l-Arg with E84. Furthermore, hydrogen bonding of the Fe-bound D191 with the NH_2_ of the l-Arg guanidium group also contributes to the substrate orientation in the active site. Principal component analysis (PCA) of the Fe(iii)-superoxo dynamics identified the main direction of motions in the enzyme. Three regions showed significant flexibility: (i) the loop connecting residues 80–93 along with β4 and β5 (ii) the loop-forming residues 211–245 along with β11, and (iii) the loop residues 291–303 joining β15 and α8. Hydrophobic residues surrounding the active site in β14 are affected by the motion of β15. Dynamic cross-correlation analysis revealed the correlated and anti-correlated motions between remote regions of EFE. The regions characterized by PCA show anti-correlated motion with each other. Substitution of E215A in region ii completely abolishes EFE's activity, signifying the role of a remote residue in controlling catalysis. Hydrophobic residues such as F250, F283, and A281 that interact with dioxygen and the C1 carboxylate of the 2OG show a positive correlation with region ii. The 2OG-stabilizing residue R171 exhibits a strong positive correlation with residues in β6 and β14. Thus, the dynamics of the Fe(iii)·superoxo·2OG·l-Arg complex revealed that the interdependency in the motion of loops and β-sheets in EFE potentially influences the active site interactions and, therefore, might be an important tool to control EFE reactivity through long-range correlations.

**Fig. 10:**
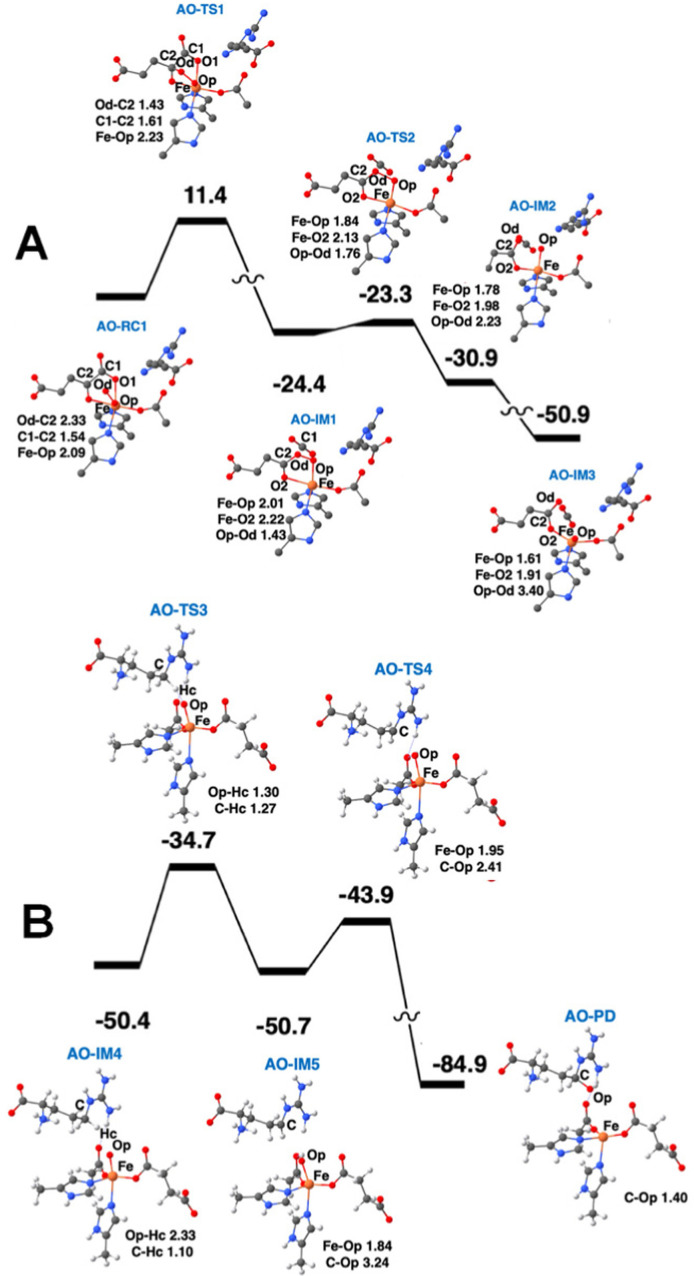
Oxygen activation and l-Arg hydroxylation mechanism of EFE. (A) The first l-Arg conformation and an off-line Fe(iii)-superoxo reaction center (labeled AO-RC1) converts to an off-line ferryl state (AO-IM3) *via* the intermediates (IM) and transition states (TS) along the potential energy surface shown. (B) After undergoing a ferryl flip to a second ferryl state (AO-IM4), HAT and hydroxyl radical rebound occur along the second potential energy surface. Hydrogen atoms and CO_2_ molecules are hidden for clarity. Bond lengths are labeled in angstroms. Energies are shown in kcal mol^−1^.

The calculated potential energy profile and QM geometries of QM/MM intermediates and transition states from one publication are displayed in Fig. 10.^38^ This figure is divided into two parts, with the oxygen activation states (the off-line Fe(iii)-superoxo state through the initial ferryl state) shown first, followed by the states involving substrate hydroxylation (the flipped ferryl intermediate through hydroxylated l-Arg). Computational studies have shown that HAT is the rate-determining step in the process of l-Arg hydroxylation. The energy barrier for the HAT reaction is 15.7 kcal mol^−1^.

The major ethylene-forming reaction catalyzed by EFE is shown on the right side of Fig. 9 and has no precedent among other enzymes.^47^ Dioxygen binds to EFE·Fe(ii)·2OG·l-Arg (state A) to form an off-line Fe(iii)-superoxo complex (not depicted) as in the case for the l-Arg hydroxylation, but the two oxygen atoms then insert into the C1–C2 bond of 2OG to generate an anhydride between carbonate and peroxysuccinate (state G). Cleavage of the O–O bond is accompanied by electron transfer from the metal center to yield a succinyl radical and carbonate coordinated to Fe(iii) (state H). Decarboxylation of the succinyl radical yields a propionate radical (state I). The propionyl radical combines with the carbonate as electron transfer restores the ferrous species (state J). This intermediate decomposes into ethylene, two carbon dioxide, and bicarbonate (state K). Further support for this mechanism is derived from an aberrant side reaction, observed using the R277K variant that fails to produce ethylene, leading to the production of 3-hydroxypropionate.^47^ This mechanistically insightful finding is highlighted in pink (state L).

Experimental and computational studies have provided valuable support for the sequence of steps forming ethylene shown in Fig. 9. For example, using 5,5-[^2^H]-l-Arg did not affect the ethylene:P5C ratio, indicating the pathways diverge before the formation of the ferryl state.^28^ The small amount of ferryl state measured by SF-UV-vis spectroscopy and Mössbauer measurements also is consistent with its association only with the pathway for the minor reaction and its absence in the ethylene forming sequence of steps. Using (3*S*,4*R*)-[^2^H_2_]-2OG, equal amounts of *cis* and *trans* 1,2-[^2^H]-ethylene was measured during ethylene formation, thus demonstrating randomization of the stereochemistry;^47^ these results support the presence of state I where the C2–C3 bond of 2OG is cleaved, allowing free rotation around the remaining C3–C4 bond. Evidence for state J includes the identification of 3-hydroxypropionate when using the R277K variant enzyme and the production of other ω-hydroxy acid products when using 2OG analogs.^47^ In addition, that publication described careful isotope tracer analysis using ^18^O_2_ that provides support for rotation of the single-^18^O-carbonate to yield partially labeled 3-hydroxypropionate and predicted bicarbonate as the initial product obtained from C1 of 2OG and an oxygen atom from O_2_.

**Fig. 11:**
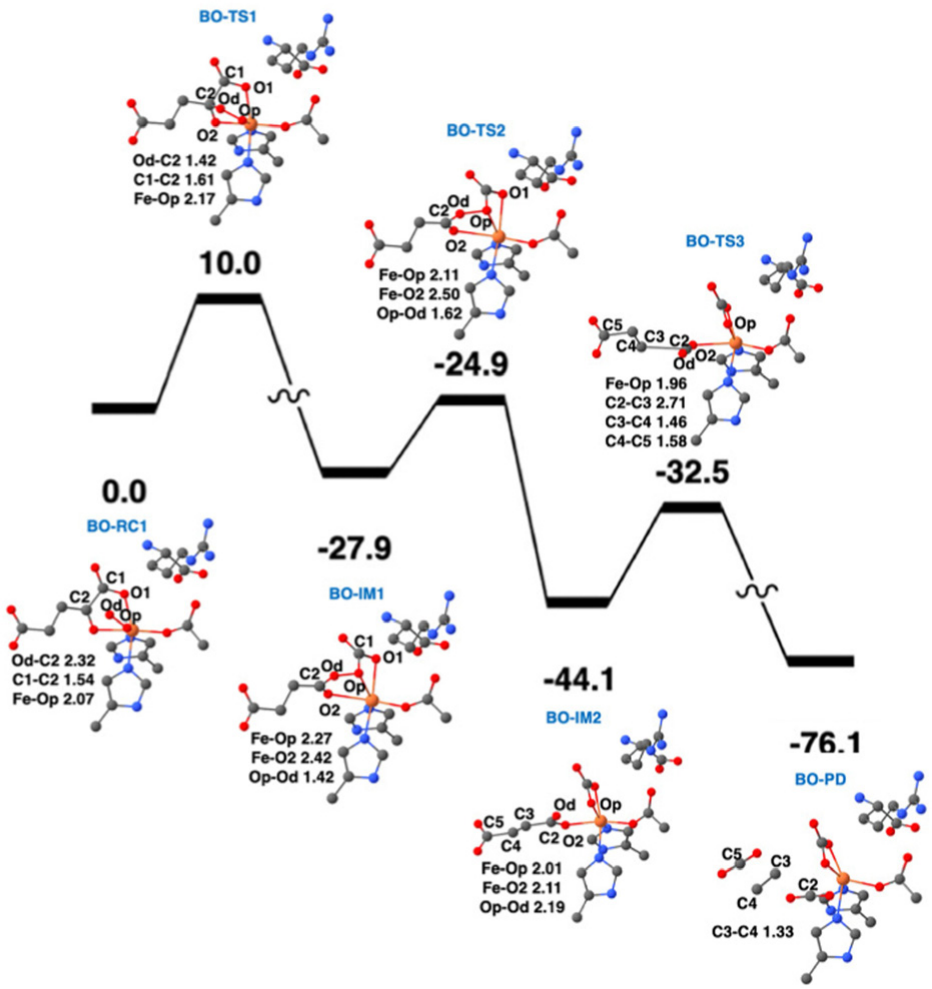
Ethylene formation mechanism of EFE. The second l-Arg conformation and an off-line Fe(iii)-superoxo reaction center (labeled BO-RC1) inserts the oxygen atoms into the 2OG C1-C2 bond (BO-IM1) forming an O-carboxy-3-hydroxypropionate (BO-IM2), that then decomposes to generate ethylene, two carbon dioxide, and ferrous ion coordinated bicarbonate along the potential energy surface shown. Bond lengths are labeled in angstroms. Energies are shown in kcal mol^−1^.^38^

QM/MM calculations from two research groups support the indicated pathway.^38,48^ The calculated reaction profile and QM geometries of QM/MM intermediates and transition states from one of these publications are displayed (Fig. 11). According to the QM/MM calculations, the first conformation of l-Arg and off-line coordination of 2OG (AO) leads to substrate hydroxylation, while the second conformation of l-Arg leads to ethylene formation by EFE. The study also explored hypothetical 2OG in-line coordination in EFE; irrespective of l-Arg conformations, hydroxylation always ensues. Molecular orbital analysis shows that the changes in electron occupancy, which are reflected in the bonding character of the Fe–O bond in the Fe(iii)-superoxo moiety by the two l-Arg conformations, determine the outcome of O_2_ activation, either forming the ferryl species that leads to hydroxylation or generating Fe(iii)-peroxycarbonate, which is hypothesized to form ethylene. In the first l-Arg conformation, the β electron in the Fe(iii)-superoxo complex resides in the π_⊥_ orbital, whereas in the second l-Arg conformation, the β electron resides in the π_‖_ orbital.

**Fig. 12:**
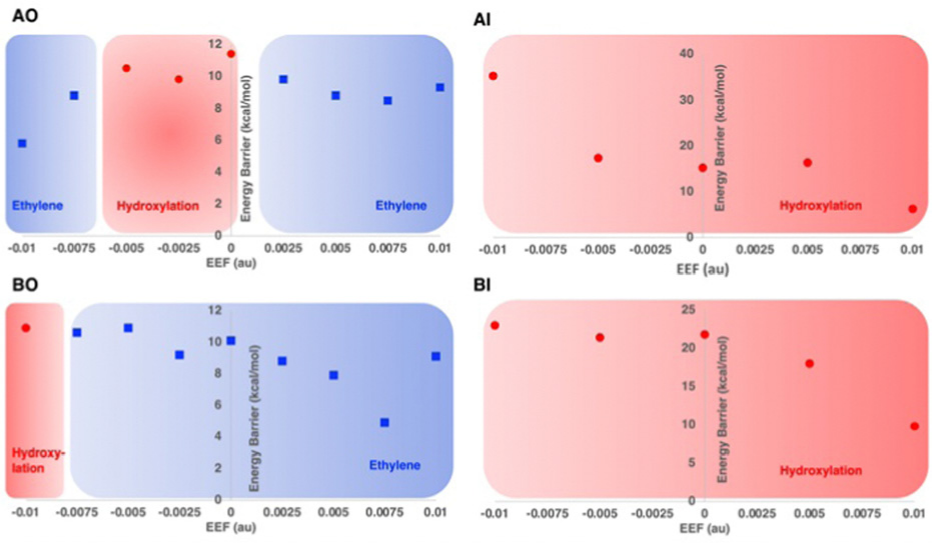
Reaction selectivities and energy barriers with respect to the strength of applied external electric field (EEF) along the Fe–O bond for different 2OG and l-Arg conformations of EFE. Reproduced from Chaturvedi *et al.* (2023).^41^

Recently, the role of the external electric field acting as a switch for the reactivity of EFE has been explored computationally.^49^ The study discussed the switch in reactivity from ethylene to hydroxylation and *vice versa* on applying different electric field intensities (Fig. 12). Based on this study, modifying the intrinsic electric field of EFE to become less negative by applying an external electric field was predicted to increase ethylene production while decreasing the amount of hydroxylation. Another computational study was based on an active site cluster of 322 atoms and suggested the two pathways diverge at the peroxysuccinate state (state B in Fig. 9), include bicarbonate radical, and lacked state J while possessing intermediates analogous to states G, H, and I.^46^ That study also explored the possibility of ethylene formation at the ferryl intermediate level and observed that energetically it is not feasible.

The critical feature(s) of EFE that uniquely allow it to generate ethylene among the large family of Fe(ii)/2OG oxygenases remain uncertain. Potential explanations have included a twist of the 2OG substrate,^26^ the D191-Y192 twisted peptide bond,^27^ the trapping of CO_2_ at the active site,^46^ the l-Arg conformations, and perhaps most importantly, an especially hydrophobic environment around the 2OG. Despite extensive computational and experimental efforts, along with preliminary mutational analyses, solid evidence is lacking to target residues for increased ethylene production from EFE. Several criteria have been utilized to achieve an improved ethylene ratio by targeting specific residues involved in l-Arg and 2OG binding. Future studies could be focused on targeting a combination of residue mutations or external factors, such as an external electric field, to improve the ethylene production of EFE.

## ACC oxidase

ACC was shown to be a precursor of plant ethylene formation more than three decades ago,^50^ and the intervening years have witnessed intensive efforts to investigate the mechanism of this intriguing ACCO enzyme.^8,51^ One intriguing early finding was that bicarbonate or carbon dioxide markedly enhanced its activity.^52^ ACCO from apple fruit was the first to be purified, with studies confirming the need for Fe(ii), carbon dioxide, and ascorbate.^53^ Although Fe(ii) is paramagnetic, electron paramagnetic resonance (EPR) studies were able to be carried out in the presence of nitric oxide (NO) that forms a paramagnetic complex with the ACCO metallocenter.^54^ In particular, use of ^15^N and ^17^O labeled D-alanine, a substrate analog of ACC, allowed for Q-band electron nuclear double resonance (ENDOR) studies that demonstrated the α-amino and α-carboxylate groups directly coordinate the metal site. Near infrared circular dichroism and magnetic circular dichroism spectroscopies were used to probe the ACCO metallocenter and revealed that the addition of CO_2_ stabilizes a six-coordinate state of the metal until ascorbate is added, whereas without CO_2_ the enzyme rapidly reacts with oxygen leading to inactivation.^55^ One possibility suggested at that time was that CO_2_ reacted with a metal-coordinated water to form Fe(ii)-bicarbonate. Single-turnover reaction studies revealed the production of substoichiometric levels of ethylene production without addition of ascorbate.^56^

**Fig. 13:**
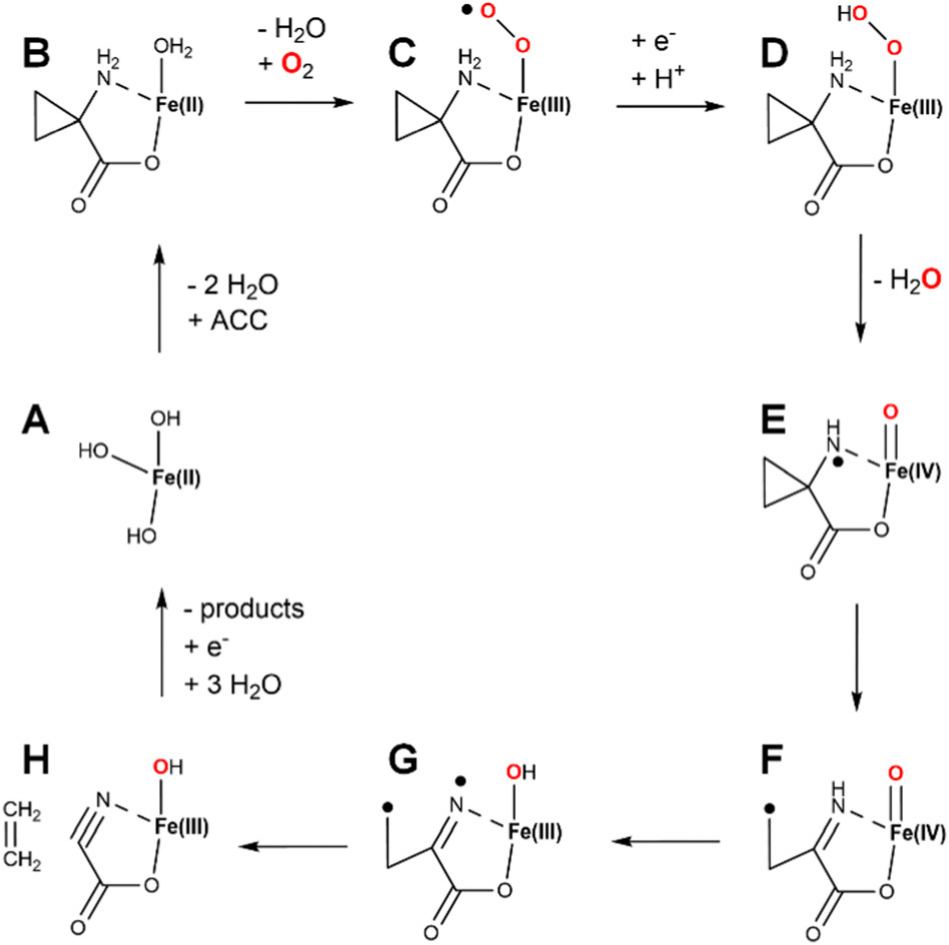
One version of a catalytic mechanism for ACCO derived from biochemical and computation studies. Binding of ACC and dioxygen accompanied by reduction and protonation yields a ferryl/N-radical intermediate (state E). Radical rearrangement and hydrogen atom abstraction provides an Fe(iii)-hydroxide/dual radical species (state G) that decomposes to form ethylene and cyanoformate (state H), with the latter molecule decomposing to cyanide and CO_2_. Reduction and product release restores the enzyme to the starting state. Ascorbic acid is used as reductant in the laboratory and may function as well in the cell. Bicarbonate (not shown) is proposed to facilitate proton transfer steps.

Fresh insight into the enzyme mechanism was obtained when the structure was solved for the enzyme (a summary of all ACCO structures is provided in Table 2).^29^ Modeling studies that utilized the structure suggested that bicarbonate bound within hydrogen bonding distance of ACC and formed electrostatic interactions with K158, R244, Y162, and R300.^57^ Investigations of alternate substrates, solvent isotope effects, and competitive oxygen kinetic isotope effects provided added additional clarity on the enzyme mechanism.^58–60^ While many early steps in catalysis were partially defined from the wealth of studies hinted above, the later mechanistic steps were still poorly understood. The complete pathway was explored by density functional theory calculations.^61^ A plausible mechanism combining aspects of the various studies is illustrated (Fig. 13). The resting enzyme (state A) binds ACC as a chelate of the metal site (state B) as two water molecules dissociate. In some versions of the mechanism, the third water molecule dissociates as dioxygen binds to form an Fe(iii)-superoxo species (state C), reduction/protonation yields an Fe(iii)-hydroxperoxide (state D), and O–O cleavage is accompanied by formation of a ferryl species and a radical on the substrate nitrogen atom (state E). An alternative version invokes reduction of Fe(ii)-dioxygen to generate an Fe(ii)-superoxo species (not shown) that transitions to an Fe(ii)-hydroperoxo/N-radical species (not shown) that forms state E.^61^ Rearrangement of state E results in C–C cleavage yielding a carbon-centered radical (state F). HAT by the ferryl species affords the Fe(iii)-hydroxide/diradical (state G) that decomposes to release ethylene while forming the labile cyanoformate molecule (state H).^62^ Product release and metal reduction restores the starting enzyme. Notably, bicarbonate (not depicted) was postulated to assist with proton transfer steps within this pathway.

**Table 2:** Structures reported for ACC oxidase

Source	Metal	Ligand(s)	Resolution (Å)	PDB Code	Ref.
*Petunia x hybrida*	None	none	2.1	1W9Y	29
*Petunia x hybrida*	Iron	Phosphate	2.55	1WA6	29
*Petunia x hybrida*	Nickel	Acetate	2.7	5TCW	Unpublished
*Petunia x hybrida*	Nickel	ACC	2.6	5TCV	Unpublished
*Arabidopsis thaliana*	Zinc	Pyrazine-2-carboxylic acid	2.1	5GJ9	64
*Arabidopsis thaliana*	Zinc	Pyridine-2-carboxylic acid	2.1	5GJA	64

**Fig. 14:**
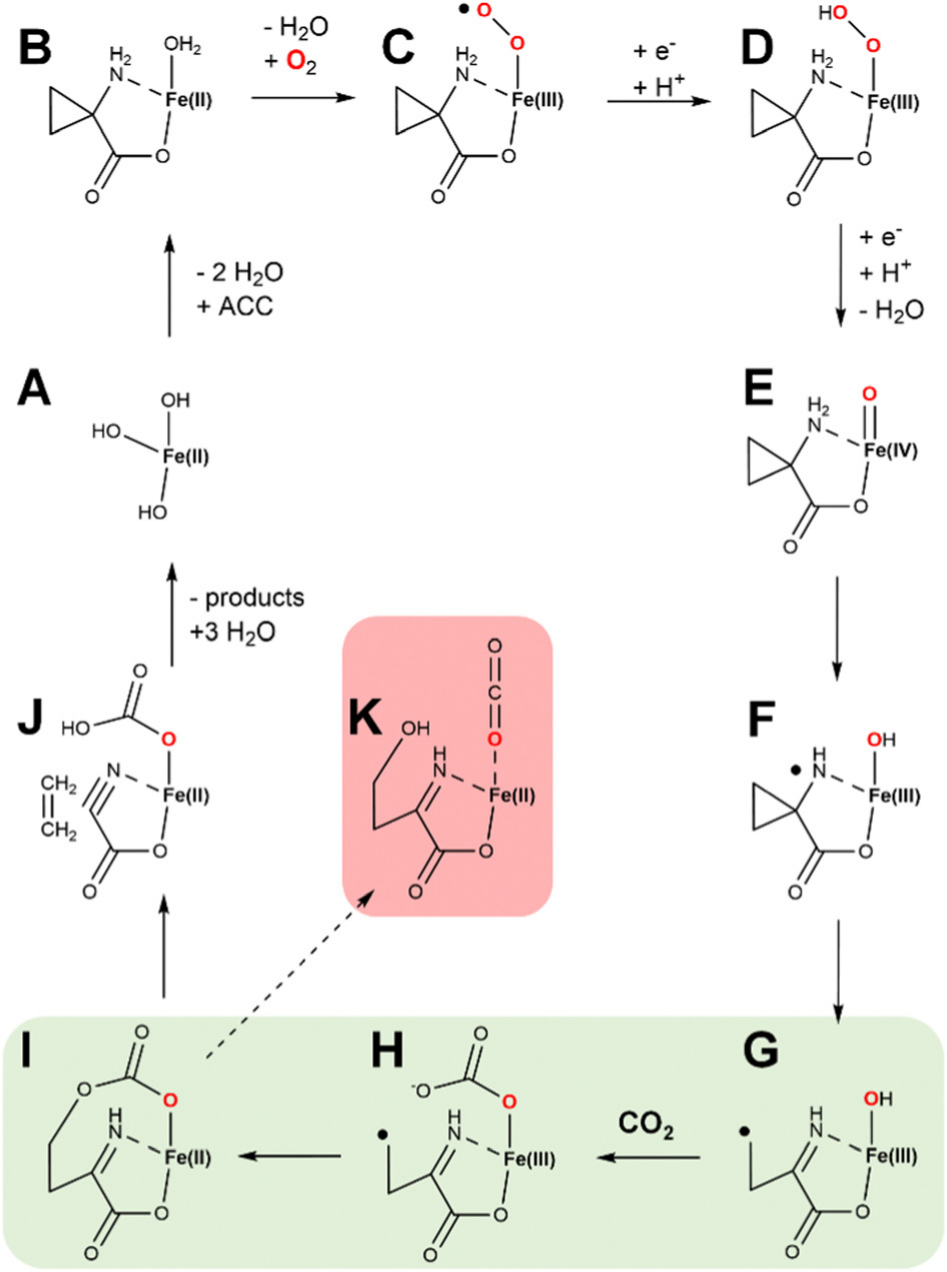
Newly proposed hypothetical enzymatic mechanism for ACCO. States A through D are equivalent to steps in Fig. 13. Newly proposed are the steps involving ferryl formation, HAT, and those highlighted in green in which a carbon centered radical is joined to the Fe(iii)-carbonate, facilitating ethylene elimination and formation of cyanoformate. The latter intermediate decomposes to hydrogen cyanide and CO_2_. Also shown (highlighted in red) is a plausible side product of the reaction.

We present a new hypothetical mechanistic proposal for ACCO that combines many of the steps described earlier with a novel role for the requisite CO_2_ (Fig. 14). States A through D are equivalent to what is shown in Fig. 13 and include one electron transfer from ascorbic acid. At this point, ferryl formation is accompanied by the second reductive step and a proton transfer (generating state E). HAT generates a nitrogen radical along with the Fe(iii)-hydroxide (state F). The substrate-derived radical rearranges with ring opening to generate a carbon centered radical (state G). We show CO_2_ binding at this point to form an Fe(iii)-carbonate (state H); however, CO_2_ binding could have occurred at an earlier step. Significantly, we suggest the carbon radical may link to the carbonate with electron transfer to the metal (state I), setting up the intermediate for elimination of ethylene (state J). The remaining cyanoformate intermediate then spontaneously decomposes^62^ to hydrogen cyanide and CO_2_. Notably, the steps shown in green highlight (states G → H → I) are reminiscent of the steps during the conversion of the propionyl radical to ethylene in Fig. 9. This novel mechanism has the potential of generating a side product (state K), that after imine hydrolysis would produce 4-hydroxy-2-oxo-butanoic acid. To the best of our knowledge, this compound has not been reported to form in ACCO reactions; however, such an aberrant product should be screened for, especially using active site variants.

## Conclusions

The two best characterized ethylene biosynthesis enzymes are EFE and ACCO. The reactions promoted by these enzymes differ greatly in their substrates (2OG *versus* ACC), their activator molecules (l-Arg *versus* CO_2_/bicarbonate), and their non-ethylene and non-CO_2_ products (succinate, guanidine, and P5C *versus* hydrogen cyanide). Nevertheless, the enzymes share many significant similarities. They are related in sequence and structure, they both require Fe(ii), and, if the ACCO mechanism shown in Fig. 14 is correct, they share similarities in mechanism. We propose that the two enzymes have the same evolutionary origin, with the function of the likely primordial Fe(ii)/2OG oxygenase being unknown. The development of EFE in bacteria and some fungi resulted in its ability to convert 2OG into ethylene. Meanwhile, the evolution of ACCO in plants and some fungi resulted in the ability to decompose ACC into ethylene and cyanide, which requires detoxification by action of widely observed plant enzyme, β-cyanoalanine synthase.^63^

## Author contributions

Conceptualization: RPH; writing – original draft: RPH; writing – review and editing: RPH, SBJSR, MGT, SC, JH, CZC; funding acquisition: RPH, JH, CZC.

## Conflicts of interest

There are no conflicts to declare.

## Supplementary Material
